# Equivalent Self-Noise Suppression of Distributed Hydroacoustic Sensing System Using SDM Signals Based on Multi-Core Fiber

**DOI:** 10.3390/s25154877

**Published:** 2025-08-07

**Authors:** Jiabei Wang, Hongcan Gu, Peng Wang, Gaofei Yao, Junbin Huang, Wen Liu, Dan Xu, Su Wu

**Affiliations:** Naval University of Engineering, Wuhan 430033, China; wangjiabei97@163.com (J.W.); tanktomb@163.com (H.G.); fly_1375@163.com (G.Y.); tsyj98@163.com (J.H.); liusenanbei@163.com (W.L.); xudan0303@163.com (D.X.); 0909042005@nue.edu.cn (S.W.)

**Keywords:** multi-core fiber, distributed hydroacoustic sensing, equivalent self-noise suppression

## Abstract

**Highlights:**

Multi-core optical fiber is applied to the field of hydroacoustic sensing. A dual-channel distributed acoustic sensing system based on multi-core optical fiber is established. The noise correlation between two cores is analyzed. The experiment verified that the space division multiplexing of the two-core signals can suppress the equivalent self-noise of the system and improve the detection performance for weak signals.

**What are the main findings?**

- The distributed acoustic sensing system based on multi-core optical fiber is applied in the field of hydroacoustic detection.
- The self-noise level of the system is suppressed by the space division multiplexing of dual-core signals.

**What is the implication of the main finding?**

- The system’s detection capability of weak hydroacoustic signals can be enhanced.
- The sensing unit meets the requirement of small size in this method.

**Abstract:**

To address the demand of equivalent self-noise suppression in a distributed hydroacoustic sensing system, this study proposes a method to enhance the acoustic sensitivity and signal-to-noise ratio (SNR) using space division multiplexed (SDM) technology based on multi-core fiber (MCF). Specifically, a dual-channel demodulation system for distributed acoustic sensing is designed using MCF. The responses of different cores in MCF are almost consistent under external acoustic pressure, while their self-noise is inconsistent. Accordingly, the acoustic pressure phase sensitivity (APPS) and SNR gain based on the accumulation of dual-channel signals are analyzed, which are verified by experiments. It is shown that the self-noise correlation coefficient between the two cores is 0.11, increasing the noise power by 3.46 dB. The APPS is increased by 5.97 dB re 1 rad/μPa after the accumulation of two-core signals, which is close to the theoretical value (6 dB). The equivalent self-noise is reduced by 2.54 dB. The experimental results reveal that the enhancement of acoustic pressure phase shift sensitivity and SNR can be achieved by the space division multiplexing (SDM) of multi-core signals, which is of great significance for suppressing the equivalent self-noise of the system and realizing the acoustic pressure detection of weak underwater signals.

## 1. Introduction

Fiber-optic distributed acoustic sensing (DAS) [1] technology involves injecting the probe pulse laser into the sensing fiber, while the returned probe light, mainly affected by the fluctuations of refractive index caused by the non-uniformity of doping and the geometric asymmetry, undergoes coherent Rayleigh backscattering (RBS) [2,3]. The strain at any section of the sensing fiber can be obtained through phase demodulation, and the acoustic signal can be restored. Thus, the sensing fiber is a distributed acoustic wave sensor [4]. Due to multiple advantages, such as compact structure, strong anti-electromagnetic interference, flexible array structure, and so on, DAS can provide an innovative solution for hydroacoustic detection.

DAS systems are usually based on single-mode fibers for sensing. RBS is extremely weak during long distance transmission [5], and the equivalent self-noise of the system is relatively high, which makes the detection of a weak acoustic signal full of challenges. In recent years, special optical fibers have been introduced to improve the performance of DAS, which include continuous scattering enhanced fiber [6,7,8,9,10], ultra-weak fiber Bragg grating (UWFBG) [11,12,13,14], scattering enhancement point (SEP) fiber [15,16], spiral winding sensitized fiber [17,18,19,20,21,22], secondary coating sensitized fiber [23], and so on. A distributed sensing system with UWFBG arrays was established [12,24] with the maximum sensitivity reaching −113 dB re 1 rad/μPa@10 to 1000 Hz [24]. Secondary coating sensitization was applied to the distributed UWFBG arrays [25,26,27], with the minimum diameter reaching 0.4 mm and the maximum sensitivity reaching −126.04 dB re 1 rad/μPa @10 Hz [26]. However, there are still problems such as uneven sensitivity response [25] and poor fidelity of low-frequency waveforms [26]. Helically wound UWFBG hydrophone [28,29] with a minimum diameter of 10 mm could reach a maximum sensitivity of −133.99 dB re 1 rad/μPa [29]. When wrapping the single-mode optical fiber on a composite thin-wall tube with a diameter of 15 mm, the average sensitivity was −129.23 dB re rad/µPa [17]. Furthermore, when the scattering enhanced fibers are helically wound on the sensitization layer [30,31], the sensitivity can reach −127 dB re rad/µPa. The increasing diameter of the hydrophone makes it challenging to apply in some situations that require small-sized hydrophones. The scattering enhancement scheme was introduced into UWFBG fiber [32,33,34,35,36], achieving a sensitivity as high as −101.21 dB re 1 rad/μPa @ 100~3000 Hz [36]. However, increases the optical power loss, which limits the detection distance. Therefore, it is necessary to explore new sensitization methods to meet the requirements of small size and long distance.

In the field of hydroacoustic sensing, the method for improving sensitivity is currently widely adopted [17,37,38,39]. The feature of multiple core sensing in parallel with MCF can not only achieve the monitoring of multiphysics [40] but also provides a new sensitization method using the accumulation of multi-core signals. The sensitivity of the system can be further enhanced by the accumulation of multiple core signals. Theoretically, the signals of multiple arrays can be superimposed in phase to improve the strength of weak signals [41,42,43], but the noise is also added together at the same time. Only improvement in the SNR is effective [44]. A variety of denoising methods are applied to improve the SNR. The internal noise of the system can be suppressed by improving the performance of the hardware and the structure of the system [45,46] but it is limited due to the high randomness of the noise. Therefore, multiple algorithms, such as modal decomposition [47,48,49], adaptive filtering [50], and neural networks [51], were adopted to further enhance the SNR. However, the result is delayed because of the complexity of the algorithm, making it unsuitable for real-time monitoring scenarios.

In this paper, a scheme through signal accumulation using MCF is proposed to suppress the equivalent self-noise of the DAS system. A dual-channel DAS demodulation system based on MCF is designed. Two cores are selected as the sensing optical fibers. The hydroacoustic signal is loaded to the sensing fiber. Since the responses of each fiber core to hydroacoustic pressure are almost consistent but the noise is not, the self-noise of the system can be suppressed by accumulating the demodulated phase signals of each core. The APPS and SNR gain characteristics of the system are verified through the vibration liquid column experiment. The results are of great significance for the DAS system to improve the detection performance of underwater weak signals. This work lays the foundation for the application of optical fiber hydrophones in real-time monitoring scenarios that require small size and long distance.

## 2. Materials and Methods

### 2.1. Dual-Channel DAS System Based on Φ-OTDR

The phase changes caused by acoustic waves can be detected through DAS system based on Φ-OTDR . When the far-field planar acoustic waves are subjected to L-long sensing fibers, strain occurs on the fiber, resulting in a phase change in the RBS signals. The relationship between the optical phase variation and fiber strain is expressed as [26]

(1) $$\Delta \varphi =\frac{4\pi n_{eff}L}{\lambda }(\frac{\Delta L}{L}+\frac{\Delta n_{eff}}{n_{eff}})$$

where $n_{eff}$ is the effective refractive index of the fiber and λ is the wavelength of the incident light. The change in index $\Delta n_{eff}$ is expressed as

(2) $$\Delta n_{eff}=-\frac{1}{2}n_{eff}^{3}(p_{11}\varepsilon_{r}+p_{12}\varepsilon_{\theta }+p_{12}\varepsilon_{z})$$

where $p_{11}$, $p_{12}$ are the elastic–optical coefficients of the fiber, $\varepsilon_{r}$, $\varepsilon_{\theta }$, and $\varepsilon_{z}$ are the radial, circumferential, and axial strains of the fiber, respectively, and $\Delta L/L=\varepsilon_{z}$.

**Figure 1:**
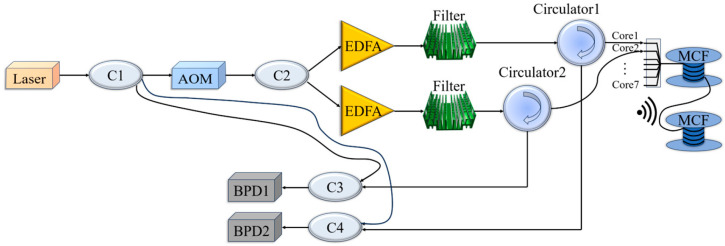
Dual-channel distributed hydroacoustic sensing system with MCF.

The principle of heterodyne coherent demodulation of a dual-channel DAS system is depicted in Figure 1. The light from the narrow linewidth laser is split by coupler C1 with a splitting ratio of 98:1:1, most of which passes through an acousto-optic modulator (AOM; the pulse frequency is 9985 Hz, and the pulse width is 200 ns) to generate an optical pulse. This is then split into two beams of transmitted light by coupler C2 (splitting ratio of 50:50) to be injected into the sensing fiber via respective erbium-doped fiber amplifiers (EDFA), filters, and circulators. The two RBS pulses interfere with the two reference lights of 1% at couplers C3 and C4 (both splitting ratios of 50:50). The interference signals are acquired by a balanced detector (BPD) and a data acquisition (DAQ) card.

**Figure 2:**
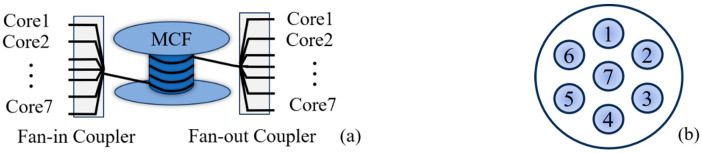
(**a**) MCF structure; (**b**) core distribution.

The structure of the 7-core fiber is shown in Figure 2a. The pigtails are connected to the single-mode fiber through fan-in/out couplers. The spatial distribution of the 7 cores is shown in Figure 2b, where core 7 is located at the center and the other 6 cores are evenly distributed around it. The outer diameter of MCF is 245 μm. The spacing between adjacent cores is consistent. Hydroacoustic pressure is applied uniformly to each core, hence the phase change in each core is consistent, which is a prerequisite for the signal to be accumulated. Cores 1 and 2 are used in the experiments.

The detected signal using BPD can be expressed as

(3) $$E=E_{0}^{2}+E_{l}^{2}+2E_{0}E_{l}\cos(2\pi \Delta ft+\varphi_{l}-\varphi_{0})$$

where $E_{0}$ is the amplitude of the light emitted by the laser, $E_{l}$ is the amplitude of the RBS light at the disturbed position l, Δf is the frequency shift generated by the AOM, $\varphi_{l}$ is the phase of the RBS light at l, and $\varphi_{0}$ is the initial phase of the light of the laser. Using the phase-demodulated method [52], the phase difference signal can be obtained as

(4) $$\Delta \varphi =\varphi_{l}-\varphi_{0}=\tan^{-1}(\frac{E_{0}E_{l}\sin(\varphi_{l}-\varphi_{0})}{E_{0}E_{l}\cos(\varphi_{l}-\varphi_{0})})$$

Then, the acoustic information can be obtained and disturbance position can be achieved.

### 2.2. Array Gain Accumulation Principle

Assuming the linear summation of signals $s_{1}(t)$, $s_{2}(t)$,⋯, $s_{m}(t)$ of m array elements with uniform sensitivity, the average power is [53]

(5) $$\bar{S^{2}}=a\bar{{\left[s_{1}(t)+s_{2}(t)+\cdots +s_{m}(t)\right]}^{2}}$$

where a represents the scaling factor. Similarly, the average noise power is

(6) $$\bar{N^{2}}=a\bar{{\left[n_{1}(t)+n_{2}(t)+\cdots +n_{m}(t)\right]}^{2}}$$

Letting $\bar{s_{1}^{2}}=\bar{s_{2}^{2}}=\cdots =\bar{s_{m}^{2}}$, $\bar{n_{1}^{2}}=\bar{n_{2}^{2}}=\cdots =\bar{n_{m}^{2}}=\bar{n^{2}}$, then the SNR can be expressed as

(7) $$\begin{array}{ll} \frac{\bar{S^{2}}}{\bar{N^{2}}} & =\frac{\bar{s^{2}}}{\bar{n^{2}}}\frac{\left[{\left(\rho_{s}\right)}_{11}+{\left(\rho_{s}\right)}_{12}+\cdots +{\left(\rho_{s}\right)}_{1m}]+\cdots +[{\left(\rho_{s}\right)}_{m1}+{\left(\rho_{s}\right)}_{m2}+\cdots +{\left(\rho_{s}\right)}_{mm}\right]}{\left[{\left(\rho_{n}\right)}_{11}+{\left(\rho_{n}\right)}_{12}+\cdots +{\left(\rho_{n}\right)}_{1m}]+\cdots +[{\left(\rho_{n}\right)}_{m1}+{\left(\rho_{n}\right)}_{m2}+\cdots +{\left(\rho_{n}\right)}_{mm}\right]}\\ & =\frac{\bar{s^{2}}}{\bar{n^{2}}}\frac{\sum_{j}\sum_{i}{\left(\rho_{s}\right)}_{ij}}{\sum_{j}\sum_{i}{\left(\rho_{n}\right)}_{ij}} \end{array}$$

where ${\left(\rho_{s}\right)}_{ij}$, ${\left(\rho_{n}\right)}_{ij}$ is the correlation coefficient of signal and noise between the i and j array elements, respectively.

(8) $${\left(\rho_{s}\right)}_{ij}=\frac{\bar{\sum s_{i}(t)s_{j}(t)}}{{\left(\bar{\sum \left(s_{i}(t\right){)}^{2}{\left(s_{j}(t)\right)}^{2}}\right)}^{1/2}}$$

(9) $${\left(\rho_{n}\right)}_{ij}=\frac{\bar{\sum n_{i}(t)n_{j}(t)}}{{\left(\bar{\sum \left(n_{i}(t\right){)}^{2}{\left(n_{j}(t)\right)}^{2}}\right)}^{1/2}}$$

The SNR gain is

(10) $$AG=10\mathrm{lg}\frac{\bar{S^{2}}/\bar{N^{2}}}{\bar{s^{2}}/\bar{n^{2}}}=10\mathrm{lg}\frac{\sum_{j}\sum_{i}{\left(\rho_{s}\right)}_{ij}}{\sum_{j}\sum_{i}{\left(\rho_{n}\right)}_{ij}}$$

Thus, the gain after array accumulation depends on $\rho_{s}$ and $\rho_{n}$. Assuming the signals are completely correlated ($\rho_{s}=1$), s(t) will be linearly accumulated and $\bar{S^{2}}=m^{2}\bar{s^{2}}$. Considering the noise is perfectly uncorrelated

(11) $$\left\{\begin{array}{l} {\left(\rho_{n}\right)}_{ij}=0,i\neq j\\ {\left(\rho_{n}\right)}_{ij}=1,i=j \end{array}\right.$$

The noise of array elements is independent of each other. $\bar{n^{2}}$ is linearly accumulated ($\bar{N^{2}}=m\bar{n^{2}}$) and the noise gain is NG=10lgm. Obviously, the SNR gain after the accumulation of m array elements is AG=10lgm. However, if $\rho_{n}$ is partially correlated

(12) $$\left\{\begin{array}{l} {\left(\rho_{n}\right)}_{ij}=\rho ,i\neq j\\ {\left(\rho_{n}\right)}_{ij}=1,i=j \end{array}\right.$$

$\bar{n^{2}}$ is nonlinearly accumulated due to the cross-term of the noise correlation coefficient ${\left(\rho_{n}\right)}_{ij}(i\neq j)$, which is $\bar{N^{2}}=[m+(m^{2}-m)\rho ]\bar{n^{2}}$ and $NG=10\log(m+(m^{2}-m)\rho )$. Then, the SNR gain is

(13) $$AG=10\mathrm{lg}\frac{m}{1+(m-1)\rho }$$

**Figure 3:**
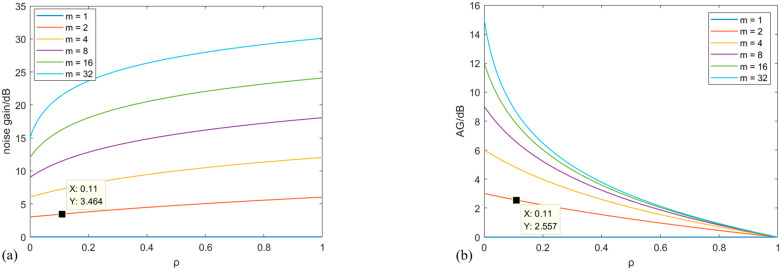
(**a**) Trend between ρ and NG at different m; (**b**) trend between ρ and AG at different m.

Obviously, the SNR gain at this point is less than 10lgm. In fact, the correlation between every two array elements may vary due to factors such as the distance between the array elements. When they are all the same, the trends between ρ and NG at different m are shown in Figure 3a. NG nonlinearly increases with the increase of ρ at the same m. In Figure 3b, when the m is fixed, the larger the ρ, the less AG. The rate of change gradually decreases. In one word, AG can be better improved with more m at an identical ρ.

## 3. Results

**Figure 4:**
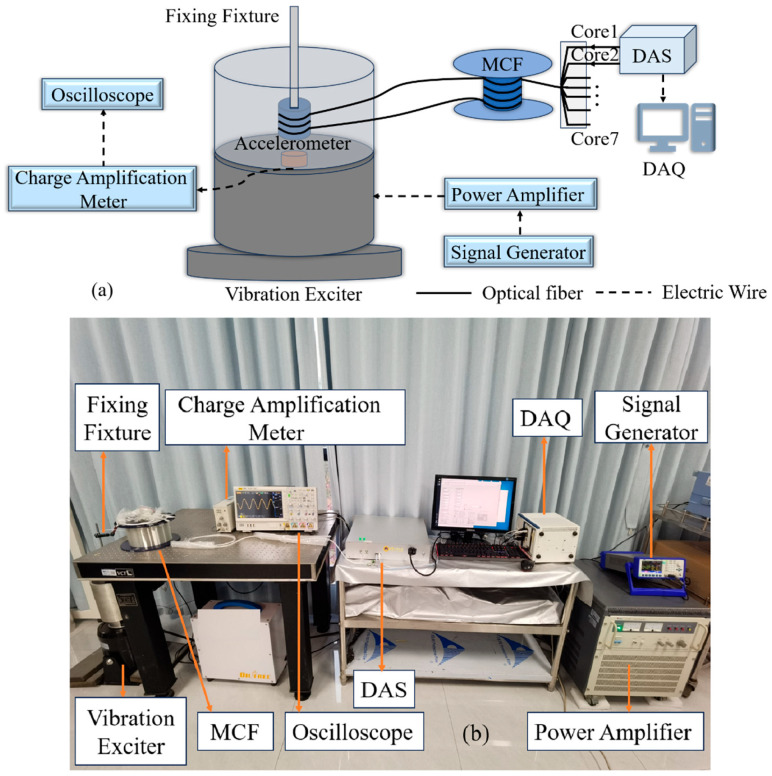
(**a**) Experimental schematic; (**b**) physical diagram of experiment.

The experiment system based on the method of a vibrating liquid column (MVLC) is shown in Figure 4a. The sinusoidal vibration signal generated by a signal generator is transmitted to the vibration exciter through a power amplifier. To verify the effectiveness on enhancing sensitivity with the accumulation of multi-core signals and guarantee the availability of MVLC, the 10 m length of MCF is uniformly loosely wound on a rigid cylinder, which is attached to the center of the vibrating liquid column by a fixture. The acoustic information captured by the sensing fiber is demodulated by the DAS module. Finally, the demodulated data is acquired by DAQ. An accelerometer is fixed at the bottom center of the vibrating liquid column tank. The output signal of the accelerometer is amplified by a charge amplifier and then displayed on an oscilloscope. The physical diagram is shown in Figure 4b.

In the experiment, the dimensions of the vibrating liquid column tank are an outer diameter of 17 cm, an inner diameter of 15.2 cm, and a height of 20 cm. The liquid is distilled water with a depth of 16 cm. The 10 m length of MCF is placed at a position of 10.5 cm underwater. The room temperature is approximately 25 degrees during the test.

### 3.1. Sensitivity Analysis

The sensitivity of the DAS system based on Φ-OTDR essentially refers to the change in light phase caused by sound pressure in the sensing fiber, which is usually expressed as the acoustic pressure phase shift sensitivity level [28]:

(14) $$M=20\mathrm{lg}(\frac{A_{\Delta \varphi }}{p_{0}})-120$$

where $A_{\Delta \varphi }$ is the amplitude of Δφ, and $p_{0}=1\mu Pa$ is the amplitude of the sound pressure. The higher the sensitivity, the more it can respond to the weak hydroacoustic signal. The equivalent self-noise of the system is determined by sensitivity and the self-noise spectral level. When there is no sound pressure at the sensitive end of the hydrophone, the equivalent self-noise of the system is the difference between the self-noise spectral level $U_{n}$ at the demodulated output of the hydrophone and the APPS level M, which can be expressed as

(15) $$L_{ps}=20\mathrm{lg}U_{n}-20\mathrm{lg}M-20\mathrm{lg}p_{0}$$

Obviously, lower $U_{n}$ and higher M are more favorable for the detection of weak signals with.

Multiple cores in MCF are independently and synchronously sensed in parallel, and each core is independently led out through fan-in/out couplers. As shown in Figure 1, two beams of pulse light, with a uniform pulse width and ignorable pulse broadening, are simultaneously injected into two equal-length cores. Therefore, the signals in the two cores at the same time are synchronized and can be accumulated directly.

**Figure 5:**
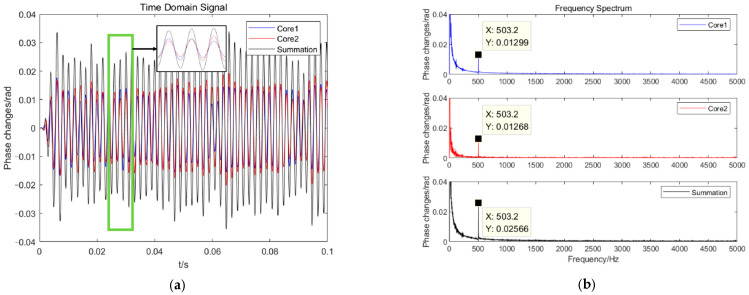
The responses of the signals at 500 Hz. (**a**) Time domain signal. (**b**) Spectral signal.

To verify the change in sensitivity before and after the accumulation of the signals, sensitivity testing experiments were carried out based on MVLC. The time domain waveforms before and after accumulation at 500 Hz are shown in Figure 5a. The signals are restored well. The phase of signals in core 1 and core 2 has great consistency. Their spectral characteristics are obtained after Fourier transformation, as shown in Figure 5b. The amplitudes are about 0.013 rad, 0.013 rad, and 0.026 rad, respectively.

**Figure 6:**
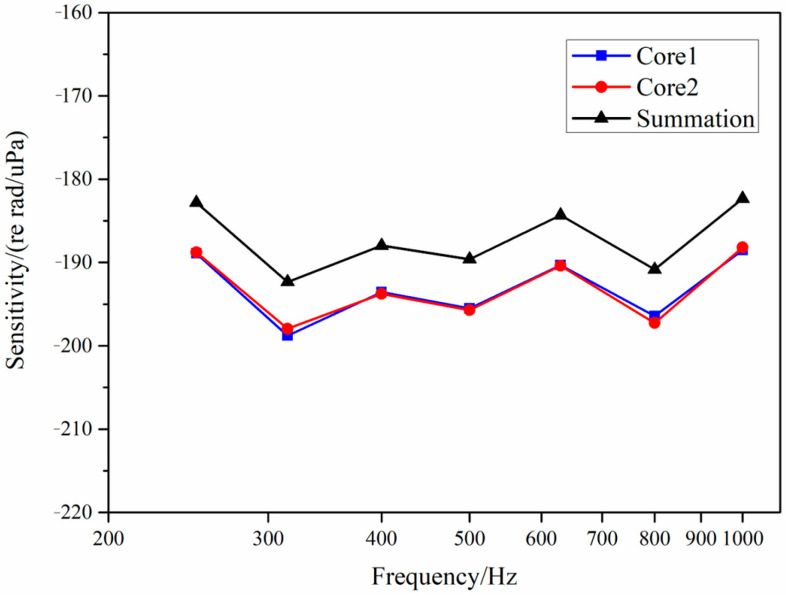
Sensitivity comparison.

The frequency response curves for sensitivity in the band of 250 Hz–1000 Hz were tested, as shown in Figure 6. The sensitivity level of the MCF has a certain fluctuation under the hydroacoustic pressure at different frequencies, ranging from −188 to −198 dB re 1 rad/μPa. The main reason is that the fiber is not tightly wound, and the MCF with only one coating layer is basically in a free state when responding to hydroacoustic pressure. The sensitivities of the two cores are almost consistent. The maximum difference is 0.85 dB. The sensitivity after the summation is steadily increased with an average of 5.97 dB, close to the theoretical value of 6 dB. The sensitivity data corresponding at each frequency in Figure 6 are shown in Table 1. The standard deviation of the gain is 0.12 dB, and its confidence interval is [5.80 6.14] dB at a confidence level of 99%.

### 3.2. Analysis of Noise Performance

**Figure 7:**
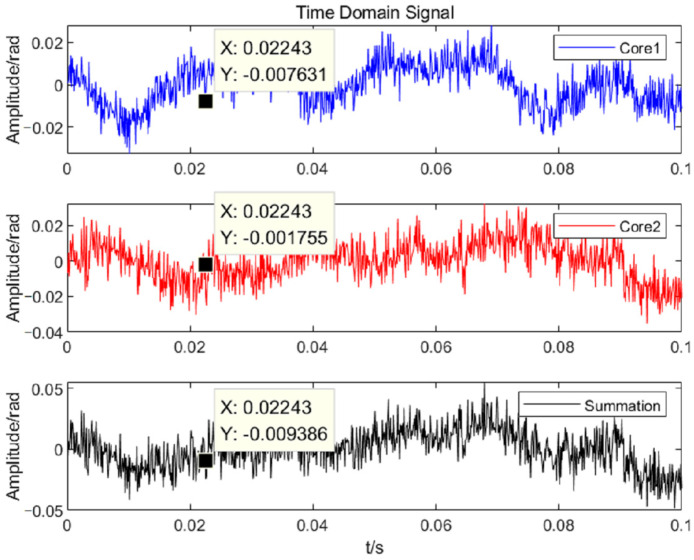
Time domain curves before and after summation of two-core noise.

**Figure 8:**
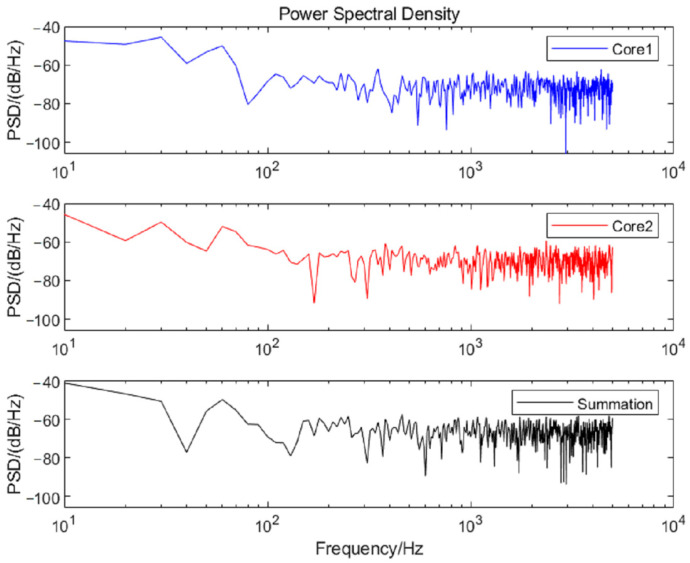
PSD before and after double-core noise accumulation.

The time domain curves and power spectral densities (PSD) of the noise for the two cores are analyzed, as shown in Figure 7 and Figure 8. According to the formula $E[n^{2}(t)]=\frac{1}{2\pi }\int_{-\infty }^{\infty }G_{n}(\omega )d\omega$ ($G_{n}(\omega )$ is the PSD of the noise), the average powers of the noise in the frequency band of 100–1000 Hz are calculated as −66.82 dB, −66.56 dB, and −63.26 dB, respectively. The results of five repetitions are shown in Table 2.

Based on the results in Section 2.2, the theoretical gain is 3 dB when the completely uncorrelated two-core noises are summed. As calculated by Equation (9), ρ is 0.11 and NG is 3.46 dB, as shown in Figure 3a. As can be seen in Table 2, the actual average NG is 3.46 dB, which is consistent with the calculated value. The standard deviation of the gain is 0.06 dB, and its confidence interval is [3.34 3.59] dB at a confidence level of 99%. From the perspective of the experimental system, the correlation of noise mainly comes from two aspects. On one hand, the noise source of the dual channels is partly from the same optical device, such as the laser and AOM. On the other hand, the responses to the noise of the identical position at cores 1 and 2 are the same. Underwater noise forms a spatially continuous sound pressure field around the optical fiber when propagating. All cores are subjected to similar sound pressure disturbances at the same position, resulting in the correlation of optical phase modulation. The mechanical vibration noise is synchronously transmitted to all cores through the shared cladding and coating of MCF. The refractive index of each core is simultaneously affected by the thermal expansion due to the temperature changes. Therefore, NG after the summation is greater than the theoretical value.

According to the structure of MCF shown in Figure 2b, ρ between two adjacent fiber cores for cores 1–6 may be the same because of the identical core spacing. The ρ decreases due to the enlarging of the core spacing. Since core 7 is located at the center of the fiber, it is less affected by environmental noise compared to other cores. Therefore, its correlation coefficient with other cores may be reduced.

### 3.3. Analysis of Equivalent Self-Noise Pressure Suppression Performance

**Figure 9:**
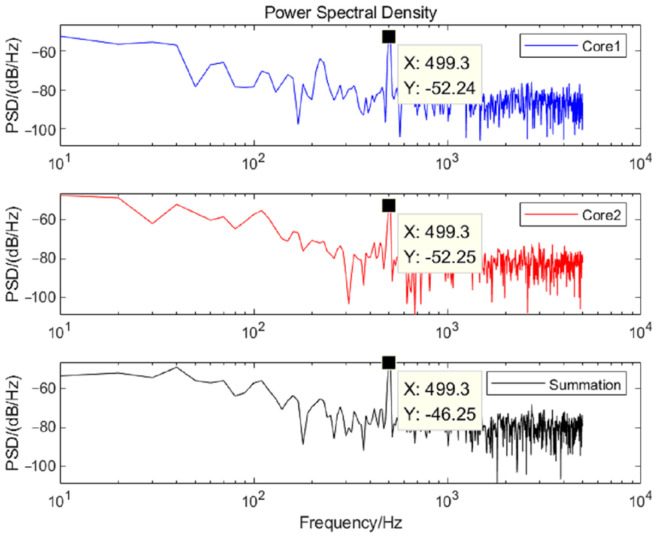
Comparison of PSD before and after accumulation of two-core signals.

To verify the equivalent self-noise suppressed after the summation, the PSD curves of the demodulated phase signals at a frequency of 500 Hz are shown in Figure 9. The PSD at 500 Hz are −52.24 dB, −52.25 dB, and −46.25 dB, respectively, with an average enhancement of about 5.99 dB, which is consistent with the theoretical derivation. The results of multiple frequencies are shown in Table 3. The standard deviation of the gain is 0.03 dB, and its confidence interval is [5.92 6.07] dB at a confidence level of 99%.

Then, AG at each frequency is further calculated. When ρ is 0.11, AG is theoretically 2.56 dB, based on Equation (13). The results of the experiment are shown in Table 4. The average AG is 2.54 dB, which is close to the theoretical value shown in Figure 3b. The standard deviation of the gain is 0.12 dB, and its confidence interval is [2.18 2.90] dB at a confidence level of 99%. The reliability of the experimental results is verified. The improvement of SNR is important for enhancing the system’s ability to detect weak signals.

**Figure 10:**
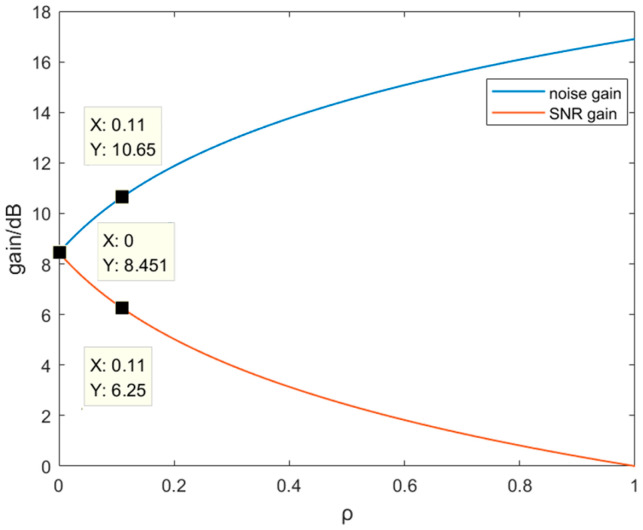
The trend between gain and ρ after the accumulation of seven-core signals.

Further, AG can reach 8.45 dB under the accumulation of seven-core signals when ρ=0, which is the ideal state, as shown in Figure 10. NG gradually increases and AG is bound to be reduced with the increase of ρ. When ρ=0.11, AG can still reach 6.25 dB.

## 4. Conclusions

In this study, MCF is applied to the field of hydroacoustic sensing. A dual-channel system of distributed hydroacoustic sensing based on heterodyne demodulation with MCF is designed. When using MCF as the sensing fiber, the responses of different cores to the sound pressure are consistent while their self-noise is inconsistent. A method of accumulating two-core signals is proposed to suppress the equivalent self-noise of the system, which is verified by experiments. The correlation coefficient of noise between two cores in the MCF is determined to be 0.11. The APPS increased by 5.97 dB re 1 rad/μPa, and the equivalent self-noise decreased by 2.54 dB, which are in line with the theoretical analysis. The SNR gain after the accumulation of seven-core signals can reach 8.45 dB through simulation when the noise is completely uncorrelated. These improvements are of great significance for the DAS system to detect underwater weak signals, e.g., seismic monitoring and oil–gas exploration.

## Figures and Tables

**Table 1:** Sensitivity at each frequency before and after accumulation (dB re 1 rad/μPa).

Frequency /Hz	Core1	Core2	Average Value	Summation	Actual Gain	Actual Average Gain
250	−188.90	−188.78	−188.84	−182.82	6.02	5.97
315	−198.78	−197.96	−198.37	−192.34	6.03	
400	−193.57	−193.76	−193.67	−187.98	5.69	
500	−195.52	−195.73	−195.63	−189.61	6.02	
630	−190.31	−190.39	−190.35	−184.33	6.02	
800	−196.42	−197.26	−196.84	−190.84	6.00	
1000	−188.51	−188.19	−188.35	−182.34	6.01	

**Table 2:** Noise PSD before and after the summation of the two-core signals (dB/Hz).

Serial Number	Core1	Core2	Summation Theoretical Value	Summation Actual Value	Actual Gain	Actual Average Gain
1	−66.81	−66.56	−63.68	−63.26	3.42	3.46
2	−66.41	−66.73	−63.57	−63.01	3.55	
3	−66.79	−66.52	−63.64	−63.18	3.46	
4	−66.47	−66.76	−63.61	−63.10	3.51	
5	−66.84	−66.61	−63.72	−63.33	3.39	

**Table 3:** PSD of each frequency before and after accumulation of two-core signals (dB/Hz).

Frequency /Hz	Core1	Core2	Average Value	Summation	Actual Gain	Actual Average Gain
500	−52.24	−52.25	−52.25	−46.25	6.00	5.99
630	−48.03	−47.77	−47.90	−41.89	6.01	
800	−56.82	−57.15	−56.99	−51.04	5.95	
1000	−38.73	−38.31	−38.52	−32.51	6.01	

**Table 4:** SNR at each frequency before and after accumulation of two-core signal (dB).

Frequency /Hz	Core1	Core2	Average Value	Summation	Actual Gain	Actual Average Gain
500	23.36	23.16	23.26	25.94	2.68	2.54
630	23.72	24.20	23.96	26.58	2.62	
800	25.49	26.20	25.85	28.24	2.39	
1000	25.03	25.10	25.07	27.52	2.45	

## Data Availability

Data underlying the results presented in this paper are not publicly available at this time but may be obtained from the authors upon reasonable request.

## References

[1] Cao W.H., Cheng G.L., Xing G.X., Liu B. (2023). Near-field target localisation based on the distributed acoustic sensing optical fiber in shallow water. Opt. Fiber Technol..

[2] Sun M.Y., Yu M., Wang H.R., Song K., Guo X., Xue S., Zhang H., Shao Y., Cui H., Chang T. (2023). Intelligent water perimeter security event recognition based on NAM-MAE and distributed optic fiber acoustic sensing system. Opt. Express.

[3] Chen D., Liu Q., He Z. (2017). Phase-detection distributed fiber-optic vibration sensor without fading-noise based on time-gated digital OFDR. Opt. Express.

[4] Si Z.P., Bu Z.H., Mao B.N., Zhao C.L., Xu B., Kang J., Li Y., Jin S.Z. (2022). Review of research on phase sensitive optical time-domain reflectometer based on phase demodulation. Laser Optoelectron. Prog..

[5] Gabai H., Eyal A. (2016). On the sensitivity of distributed acoustic sensing. Opt. Lett..

[6] Westbrook P.S., Feder K.S., Ortiz R.M., Kremp T., Monberg E.M., Wu H., Simoff D.A., Shenk S. (2017). Kilometer length low loss enhanced back scattering fiber for distributed sensing. Proceedings of the 2017 25th Optical Fiber Sensors Conference (OFS).

[7] Jason J., Popov S.M., Butov O.V., Chamorovskiy Y.K., Golant K.M., Fotiadi A.A., Wuilpart M. Sensitivity of high Rayleigh scattering fiber in acoustic/vibration sensing using phase-OTDR. Proceedings of the Optical Sensing and Detection V.

[8] Westbrook P.S., Kremp T., Feder K.S., Ko W., Monberg E.M., Wu H., Simoff D.A., Ortiz R.M. (2018). Improving Distributed Sensing with Continuous Gratings in Single and Multi-core Fibers. Proceedings of the Optical Fiber Communication Conference.

[9] Handerek V.A., Karimi M., Nkansah A., Yau A., Westbrook P.S., Feder K.S., Ortiz R.M., Kremp T., Monberg E.M., Wu H. (2018). Improved Optical Power Budget in Distributed Acoustic Sensing Using Enhanced Scattering Optical Fiber. Proceedings of the Optical Fiber Sensors. Lausanne Switzerland.

[10] Feng S.W., Xu T.W., Huang J.F., Yang Y., Li F., Zhou J.M., Yu H. (2018). Enhanced SNR phase-sensitive OTDR system with active fiber. Proceedings of the Fiber Optic Sensing and Optical Communication.

[11] Zhu F., Zhang Y.X., Xia L., Wu X.L., Zhang X.P. (2015). Improved Φ-OTDR sensing system for high-precision dynamic strain measurement based on ultra-weak fiber Bragg grating array. J. Light. Technol..

[12] Wang C., Shang Y., Liu X.H., Wang C., Yu H.-H., Jiang D.-S., Peng G.-D. (2015). Distributed OTDR interferometric sensing network with identical ultra-weak fiber Bragg gratings. Opt. Express.

[13] Sun F.A.Q.Z., Zhang W., Liu T., Yan Z.J., Liu D.M. Wideband fully-distributed vibration sensing by using UWFBG based coherent OTDR. Proceedings of the Optical Fiber Communication Conference.

[14] Nadav A., Moshe T., Avishay E. (2024). Fiber-Optic Sensor Array for Distributed Underwater Ultrasound Sensing. J. Light. Technol..

[15] Guan H., Han B., Han Z.W., Wang W., Ran Z., Yan G., Gong Y., Rao Y.-J. High performance DAS-based optical fiber hydrophone. Proceedings of the Asia Communications and Photonics Conference.

[16] Shang Y., Wang C., Ni J.S., Zhao W.-A., Li C., Cao B., Huang S., Wang C., Peng G.-D. (2019). Discussion on the sensitivity of optical cables based on distributed acoustic sensing. Opt. Rev..

[17] Yan G.F., Wang D.L., Long J.Q., Jiang L., Gong Y., Ran Z., Rao Y. (2023). High-performance towing cable hydrophone array with an improved ultra-sensitive fiber-optic distributed acoustic sensing system. Opt. Express.

[18] He X.G., Wen P.F., Yang H., Gu L.J., Lu H.L., Zhang M. (2022). Marine towing cable seismic acquisition with small trace interval based on distributed optical fiber sensing. Geophys. Prospect. Pet..

[19] Yan G.F., Long J.Q., Jiang L., Zhang M., Wang D., Rao Y. High performance marine towing cable system based on ultra-sensitive fiber-optic distributed acoustic sensing. Proceedings of the 2022 Asia Communications and Photonics Conference (ACP).

[20] Liu X., Wang C., Shang Y., Wang C., Zhao W.A., Peng G.D., Wang H.Z. (2017). Distributed acoustic sensing with Michelson interferometer demodulation. Photonic Sens..

[21] Yang Y., Xu T., Feng S., Huang J., Fang L. (2018). Optical fiber hydrophone based on distributed acoustic sensing. Fiber Opt. Sens. Opt. Commun..

[22] Yan B.Q., Zhang K.Q., Li H., Xiao X.P., Chen J.F., Fan C.Z., Yan Z.J., Sun Q.Z. (2023). Acoustic Field Imaging of Pipeline Turbulence for Noninvasive and Distributed Gas Flow Measurement. IEEE Sens. J..

[23] Lavrov V.S., Plotnikov M.Y., Aksarin S.M., Efimov M.E., Shulepov V.A., Kulikov A.V., Kireenkov A.U. (2017). Experimental investigation of the thin fiber-optic hydrophone array based on fiber Bragg gratings. Opt. Fiber Technol..

[24] Li Z.Y., Wang C.J., Gui X., Fu X.L., Wang Y.M., Wang Z., Wang Y.B. (2023). A high-performance fiber-optic hydrophone for large scale arrays. J. Light. Technol..

[25] Pang Y.D. (2020). Research on Key Technology of Ultra-Fine Line Fiber Optic Hydrophone Based on Grating Array of Wire Drawing Tower. Ph.D. Thesis.

[26] Ding P., Huang J.B., Yao G.F., Gu H.C., Liu W., Tang J.S. (2021). Weak reflection fiber Bragg grating hydrophone with secondary coating sensitization. Chin. Lasers.

[27] Ding P., Huang J.B., Pang Y.D., Zhou C.M., Gu H.C., Tang J.S. (2021). A towed line array with weak fiber Bragg grating hydrophones. Acta Photonica Sin..

[28] Wu S., Huang J.B., Pang Y.D., Wang J.B., Gu H.C. (2024). Direction-Finding Study of a 1.7 mm Diameter Towed Hydrophone Array Based on UWFBG. Sensors.

[29] Gu H.C., Wang J.B., Wang P., Zhu M., Yao G.F., Lv J.Q. (2025). Directivity Analysis and Test of a Distributed Weak Reflection Fiber Bragg Grating Linear Hydrophone Array. Acta Armamentar.

[30] Chen J.F., Li H., Liu T., Fan C.Z., Yan Z.J., Sun Q.Z. (2021). Fully distributed hydroacoustic sensing based on lightweight optical cable assisted with scattering enhanced fiber. Proceedings of the 2021 Optical Fiber Communications Conference and Exhibition (OFC).

[31] Chen J.F., Li H., Xiao X.P., Fan C.Z., Wen P.F., Yan Z.J., Sun Q.Z. (2022). Fully continuous fiber-optic hydrophone streamer with small channel spacing for marine seismic acquisition. Proceedings of the 27th International Conference on Optical Fiber Sensors.

[32] Lv Y.J., Xiao X.P., Yang Z.Y., Li H., Yan Z.J., Sun Q.Z. (2023). High spatial resolution and large measurement range strain sensor based on special fiber OFDR system. Proceedings of the 2023 Conference on Lasers and Electro-Optics.

[33] Xiao X.P., Song Q.G., Zhao W.L., Li H., Sun Q.Z., Yan Z.J. (2023). Hybrid coding ultra-weak fiber Bragg grating (UWFBG) array for high spatial resolution temperature sensing. Proceedings of the 2023 Optical Fiber Communications Conference and Exhibition.

[34] Fan C.Z., Xiao X.P., Li H., Zhao W.L., Yan Z.J., Sun Q.Z. (2023). Full link SNR equalization DAS system over 80 km based on gradient discrete scattering enhanced fiber. Proceedings of the 2023 Optical Fiber Communication Conference.

[35] Liu D.M., He T., Xu Z.J., Sun Q.Z. (2020). New type of microstructure fiber distributed acoustic sensing technology and its applications. J. Appl. Sci..

[36] Fang J., Li Y.W., Ji P.N., Wang T. (2023). Drone detection and localization using enhanced fiber-optic acoustic sensor and distributed acoustic sensing technology. J. Light. Technol..

[37] Chen J.F., Li H., Xiao X.P., Fan C.Z., Yan B.Q., Zhang S.X., Liu H.G., Ai K., Yan Z.J., Sun Q.Z. (2023). Fully distributed hydroacoustic sensing based on ultra-highly sensitive and lightweight fiber-optic hydrophone cable. Opt. Lasers Eng..

[38] Lu B., Wu B.Y., Gu J.F., Yang J.Q., Gao K., Wang Z.Y., Ye L., Ye Q., Qu R.G., Chen X.B. (2021). Distributed optical fiber hydrophone based on Φ-OTDR and its field test. Opt. Express.

[39] Zhou Y., Zhao Y., Song C., Wang J., Xu W., Li Z. (2024). GA-BP-Based Low-Noise FBG Hydroacoustic Monitoring System with Reference Sensor. Sensors.

[40] Feng C., Niu H., Wang H., Wang D.H., Wei L.X., Ju T., Yuan L.B. (2024). Probe-Type Multi-Core Fiber Optic Sensor for Simultaneous Measurement of Seawater Salinity, Pressure, and Temperature. Sensors.

[41] Gu J.F., Lu B., Yang J.Q., Wang Z.Y., Ye L., Ye Q., Qu R.H., Cai H.W. (2021). Distributed acoustic sensing based on multi-core fiber. Acta Opt. Sin..

[42] Xiao Y.H., Liu H.H., Li J.L., Shen X.L., Zhao Z.Y., Dang H., Zou D.F., Zhang A.Y., Wang P.H., Zhao Z.Y. (2024). Fading suppression and noise reduction of a DAS system integrated with multi-core fiber. Opt. Express.

[43] Liu X., Wang R.H., Chen F.Y., Qiao X.G. (2023). Sensitivity enhancement of interferometric fiber-optic accelerometers using multi-core fiber. Opt. Laser Technol..

[44] Turov A.T., Barkov F.L., Konstantinov Y.A., Korobko D.A., Lopez Mercado C.A., Fotiadi A.A. (2023). Activation Function Dynamic Averaging as a Technique for Nonlinear 2D Data Denoising in Distributed Acoustic Sensors. Algorithms.

[45] Yu Z., Lu Y., Hu X., Zhou M. (2017). Polarization dependence of the noise of phase measurement based on phase-sensitive OTDR. J. Opt..

[46] Zabihi M., Chen Y.S., Zhou T., Liu J.X., Shan Y.Y., Meng Z., Wang F., Zhang Y.X., Zhang X.P., Chen M.M. (2019). Continuous Fading Suppression Method for Φ-OTDR Systems Using Optimum Tracking over Multiple Probe Frequencies. J. Light. Technol..

[47] Qin Z.G., Chen H., Chang J. (2017). Signal-to-noise ratio enhancement based on empirical mode decomposition in phase-sensitive optical time domain reflectometry systems. Sensors.

[48] Peng H.T., Wang M.Q., He W.B., Lv X.M., Li X. (2024). Φ-OTDR denoising Algorithm Based on Empirical Mode Decomposition and Butterworth Filtering. Adv. Lasers Optoelectron..

[49] Mao B., Bu Z., Xu B., Gong H.P., Li Y., Wang H.L., Kang J., Jin S.Z., Zhao C.L. (2022). Denoising method based on VMD-PCC in cp-OTDR system. Opt. Fiber Technol..

[50] He H.J., Shao L.Y., Li H.C., Pan W., Luo B., Zou X.H., Yan L.S. (2017). SNR Enhancement in Phase-Sensitive OTDR with Adaptive 2-D Bilateral Filtering Algorithm. IEEE Photonics J..

[51] Li S.C., Liu K., Jiang J.F., Xu T.H., Ding Z.Y., Sun Z.S., Huang Y.L., Xue K., Jin X., Liu T.G. (2022). An Ameliorated Denoising Scheme Based on Deep Learning for Φ-OTDR System with 41-km Detection Range. IEEE Sens. J..

[52] Fang G.S., Xu T.W., Feng S.W., Li F. (2015). Phase-sensitive optical time domain reflectometer based on phase-generated carrier algorithm. J. Light. Technol..

[53] Urick R.J. (1990). Principle of Underwater Sound.

